# Treatment of moderate to severe restless legs syndrome: 2-year safety and efficacy of rotigotine transdermal patch

**DOI:** 10.1186/1471-2377-10-86

**Published:** 2010-09-28

**Authors:** Birgit Högl, Wolfgang H Oertel, Karin Stiasny-Kolster, Peter Geisler, Heike Beneš, Diego García-Borreguero, Claudia Trenkwalder, Werner Poewe, Erwin Schollmayer, Ralf Kohnen

**Affiliations:** 1 Department of Neurology, Innsbruck Medical University, Innsbruck, Austria; 2 Department of Neurology, Philipps University, Marburg, Germany; 3 Department of Psychiatry, University Hospital Regensburg, Regensburg, Germany; 4 Somni bene Institute for Clinical Research and Sleep Medicine, Schwerin, and Department of Neurology, University of Rostock, Rostock, Germany; 5 Sleep Research Institute, Madrid, Spain; 6 Paracelsus-Elena Hospital, Kassel, Germany; 7 Department of Clinical Neurophysiology, Georg-August University Göttingen, Göttingen, Germany; 8 Schwarz Biosciences GmbH, a member of the UCB Group of Companies, Monheim, Germany; 9 ReSearch Pharmaceutical Services Inc, Fort Washington, PA, USA; 10 Psychology Department, University of Erlangen-Nuernberg, Nuernberg, Germany

## Abstract

**Background:**

Rotigotine is a unique dopamine agonist with activity across D1 through D5 receptors as well as select adrenergic and serotonergic sites. This study reports the 2-year follow-up safety and efficacy data of an ongoing open-label multicenter extension study (NCT00498186) of transdermal rotigotine in patients with moderate to severe restless legs syndrome (RLS).

**Methods:**

Patients received a once-daily patch application of an individually optimized dose of rotigotine between 0.5 mg/24 h to 4 mg/24 h. Safety assessments included adverse events (AEs) and efficacy was measured by the International RLS Study Group Severity Rating Scale (IRLS), RLS-6 scales and Clinical Global Impression (CGI). Quality of life (QoL) was measured by QoL-RLS.

**Results:**

Of 310 patients who completed a 6-week placebo-controlled trial (SP709), 295 (mean age 58 ± 10 years, 66% females) were included in the open-label trial SP710. 64.7% (190/295 patients) completed the 2-year follow-up; 29 patients discontinued during the second year. Mean daily rotigotine dose after 2 years was 2.93 ± 1.14 mg/24 h with a 2.9% dose increase from year 1. Rotigotine was generally well tolerated. The rate of typical dopaminergic side effects, nausea and fatigue, was low (0.9% and 2.3%, respectively) during the second year; application site reactions were frequent but lower than in year 1 (16.4% vs. 34.5%). The IRLS total score improved from baseline of SP709 (27.8 ± 5.9) by 17.2 ± 9.2 in year 2 completers. Similar improvements were observed in RLS-6 scales, CGI scores and QoL-RLS. The responder rate in the CGI change item 2 ("much" and "very much" improved) was 95% after year 2.

**Conclusions:**

Transdermal rotigotine is an efficacious and well-tolerated long-term treatment option for patients with moderate to severe RLS with a high retention rate during 2 years of therapy.

**Trial registration:**

NCT00498186

## Background

Idiopathic restless legs syndrome (RLS) is a common, chronic, and often underdiagnosed neurological sensorimotor disorder with detrimental effects on sleep, daytime functioning, and quality of life [1-3]. If RLS is severe and impairs activities of daily life, a long-term therapy has to be started and maintained over years or even life-time in numerous patients. Recent guidelines and practice recommendations favor the use of non-ergot dopamine agonists over the dopaminergic agent levodopa as first-line treatment for moderate to severe RLS [4-6]; however, only a few prospective trials investigated longer-term efficacy of open-label treatment with non-ergot dopamine agonists for 6 months [7] or 1 year [8]. No data have yet been published that report on stability of treatment efficacy, progress of tolerability problems, requirements for dosages of treatments, treatment complications such as augmentation or tolerance, or the overall retention rate on treatment during longer time periods. Here, we report the long-term efficacy and safety results of an ongoing trial after 2 years of rotigotine treatment.

The non-ergot dopamine agonist rotigotine shows activity at D_1 _through to D_5 _receptors and at selected serotonergic and adrenergic receptors [9]; it has been formulated as a silicone-based transdermal patch for once-daily application to provide continuous drug delivery and stable plasma concentrations over a 24-h period. Rotigotine transdermal patch has been successfully used in the treatment of Parkinson's disease (PD) [10,11].

The transdermal delivery of a dopamine agonist is a new treatment option for RLS. Proof-of-concept for the rotigotine patch in patients with moderate to severe, idiopathic RLS was successfully demonstrated in a 1-week pilot trial [12], and efficacy and good tolerability was shown in a 6-week dose-finding trial [13] and in two 6-month randomized, placebo-controlled, fixed-dose trials conducted in Europe and the US [14,15]. To assess long-term rotigotine safety and efficacy, patients completing the dose-finding trial [13] were offered the possibility of entering an open-label extension. Data from the initial placebo-controlled trial [13] and from the first year of the open-label follow-up [16] showed stable, clinically relevant improvements in all efficacy measures.

## Methods

### Trial design

This multicenter, multinational, single-arm, open-label extension trial (NCT00498186) started in July 2003 in 33 hospital outpatient units, sleep centers, or private neurology practices in three European countries (Austria, Germany, and Spain) and is still ongoing. It is being conducted according to the Declaration of Helsinki, Good Clinical Practice, and local regulations in each country. The trial protocol and amendments were reviewed and approved by a central institutional review board in Germany (Kommission für Ethik in der ärztlichen Forschung im Fachbereich Humanmedizin der Philipps-Universität Marburg) and in Austria (Ethikkommission der Medizinischen Universität Innsbruck). Review and approval was provided by regional institutional review boards in Spain. Written informed consent was obtained from all subjects prior to inclusion in the trial.

The trial design was described in the presentation of the first year trial data [16]. Briefly, following wash-out of the double-blind medication, rotigotine was up-titrated from a starting dose of 0.5 mg/24 h to a maximum dose of 4 mg/24 h according to the individual requirements of the patients with intermediate steps of 1 mg/24 h, 2 mg/24 h, or 3 mg/24 h. The patch was administered once-daily in the morning using rotating application sites within 14 days of treatment. During the trial, patients remained on their optimum dosage; however, dose adjustments were allowed for efficacy or tolerability reasons at the discretion of the investigators. Patients were withdrawn if higher doses than the maximum of 4 mg/24 h rotigotine were required. Visits throughout the maintenance phase were scheduled at 3-monthly intervals during the second trial year. In the event of premature discontinuation, a 1-week taper period and a 2-week surveillance period for safety follow-up was planned.

### Outcome measures

Safety and tolerability were determined by adverse event (AE) documentation, patch application site assessment, changes in vital signs, body weight, 12-lead electrocardiogram (ECG), and safety laboratory parameters throughout the second trial year. 'Global rating of tolerability by the subject' was assessed using a 5-point scale (1 = 'very good' to 5 = 'very bad'); Clinical Global Impressions (CGI) item 4 'side effects' (1 = none to 4 = outweighs therapeutic effect) [17] and the Epworth Sleepiness Scale (ESS) [18] were also evaluated. In addition, the rate of patients who completed the entire 2 years of long-term treatment (retention rate), as well as the rate and reasons of those patients who prematurely withdrew from the trial, were assessed. Patch adhesiveness was also evaluated using a patient questionnaire.

When the study was commenced, no validated tool for diagnosis and severity assessment for augmentation was available. Augmentation, could, however, be reported as a reason for dose adjustment.

Efficacy was evaluated using the total score of the International RLS Study Group Severity Rating scale (IRLS) [19], the RLS-6 scales to assess severity of RLS symptoms at different time periods, quality of sleep and daytime sleepiness [20], and the CGI to assess severity of symptoms, global change of condition, and therapeutic effect [17]. In addition to the analysis of changes from baseline in these variables, treatment responders were defined by a score improvement of at least 50% for the IRLS total score or CGI-1 at the end of the 2-year maintenance phase compared with baseline; a CGI-2 responder had a rating of 'much' or 'very much improved' at the end of year 2. A remitter was defined by an IRLS score of 10 or less at the end of year 2; the proportion of patients presenting with no symptoms at the end of year 2 (IRLS score = 0) was also calculated (symptom free). 'Global rating of efficacy by the subject' was assessed using a 5-point scale ('very good' to 'very bad') and quality of life (QoL) was evaluated with the total score of the QoL-RLS questionnaire [21].

### Patients

Patients completing the double-blind phase of the preceding dose-finding trial [13] were offered participation in this open-label extension. They were not permitted to participate if serious adverse events (SAEs) were ongoing that were suspected to be related to the previous trial medication, if severe application site disorders had occurred, or if they had not been compliant during the double-blind trial. A full description of the inclusion and exclusion criteria to enter the preceding trial can be found elsewhere [13].

### Statistical analysis

The present paper reports the 2-year interim data analysis of a 5-year trial. By nature of an interim analysis, some of the end of year 1 data in the present paper may differ slightly from the published year 1 analysis [16].

All patients treated with at least one dose of trial medication were included in the safety analysis; patients analyzed for efficacy had to have at least one efficacy value during the long-term extension trial (intention-to-treat [ITT]). Analyses included changes in variables from baseline to end of year 2 and changes from end of year 1 to end of year 2. If not otherwise stated, the baseline data of the preceding dose-finding trial for all patients entering the open-label trial (n = 295) were used [13]. All variables were analyzed descriptively based on observed cases per visit; the use of the 'Last Observation Carried Forward' (LOCF) approach for some efficacy variables is indicated with the data. With respect to the validity of the efficacy variables, only those measures that were taken within 2 days of the last patch application were used.

## Results

Of a total of 295 patients entering the open-label trial, 220 patients completed the first trial year (retention rate 74.6%) and 191 patients completed the second trial year resulting in an overall retention rate of 64.7% after 2 years of treatment.

Mean age of the study population (n = 295) was 58.3 ± 10.1 years (range 22-75) at baseline with 66% of female gender. Patients had a long history of RLS symptoms; a large proportion had received pretreatment with dopaminergic drugs [13]. Baseline values of the IRLS total score (27.8 ± 5.9) indicated severe RLS on average; mean severity of daytime symptoms at rest in the RLS-6 scales was 4.9 ± 2.6 (range 0 = no symptoms, 10 = very severe symptoms).

Eighty-seven percent of the patients completing year 1 also completed year 2. Reasons for premature discontinuation during titration, the first, and second trial year are summarized in Table 1.

**Table 1:** Reasons for premature discontinuation

	Number of patients (%)		
	Titration (n = 295)	Year 1 (n = 290)	Year 2 (n = 220)
Patients withdrawn	5 (1.7)	70 (24.1)	29 (13.2)
Owing to			
Adverse event	4 (1.4)	47 (16.2)	16 (7.3)
Withdrawal of consent	1 (0.3)	6 (2.1)	2 (0.9)
Lack of efficacy	0	10 (3.5)	7 (3.2)
Protocol deviation	0	4 (1.4)	0
Unsatisfactory compliance	0	2 (0.7)	2 (0.9)
Lost to follow-up	0	1 (0.3)	0
Other reasons	0	0	2 (0.9)

### Treatment

The mean duration of rotigotine exposure was 556 ± 256 days (median 700 days). The majority of the patients (94%) were compliant during maintenance (defined as ≥85% and ≤115% application of the planned number of patches). Withdrawal for noncompliance was documented for two patients in year 1 and two patients in year 2.

**Figure 1:**
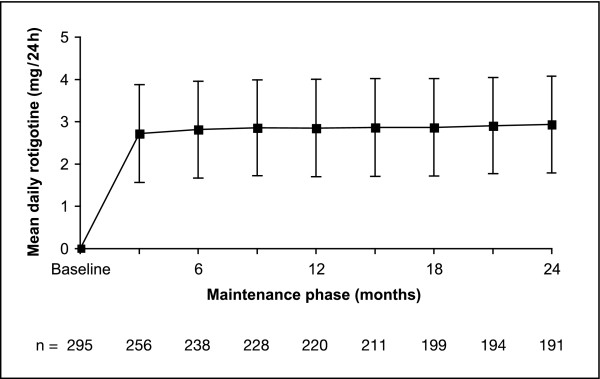
**Mean daily rotigotine dose over the 2-year maintenance period (safety population)**.

Individually optimized flexible doses ranged from 0.5 mg/24 h (2.5 cm^2 ^patch) to 4 mg/24 h (20 cm^2 ^patch). The most frequently applied dose at the end of year 2 was 4 mg/24 h (44.5% of all patients). The number of patients sufficiently treated with a dose of 0.5 or 1 mg/24 h (13.5%) decreased by 1.3% from year 1. The average daily dose during the maintenance phase showed a slight increase from 2.85 ± 1.15 mg/24 h in year 1 to 2.93 ± 1.14 mg/24 h in year 2. Figure 1 shows the mean daily rotigotine dose over 24 months of maintenance. Dose adjustment was not required for 43.8% of patients during the 2-year maintenance; doses were adjusted once in 29% and twice in 16.9% of the patients. The remaining 10.3% needed 3 to 5 dose adjustments during maintenance. Of all patients entering year 2, 88% maintained their dose during year 2.

### Safety outcomes

During the trial, 256 patients (87%) experienced at least one AE. The majority of AEs were mild or moderate in intensity; events were rated as severe in 22% of the patients.

Adverse events were documented for 75.2% of the patient population during the first trial year [16] and for 59.6% during the second year. Table 2 summarizes all incidences reported in more than 3% of all patients over the 2-year period. Application site disorders occurred most frequently; they were considered severe for 8.8% of the patients. More patients were affected by application site reactions during the first trial year (n = 100, 34.5%) whereas 16.4% (n = 36) reported them in the second year (25 patients [8.5%] had application site reactions ongoing from the preceding dose-finding study [13]). Discontinuation owing to any application site disorder was reported for 9.3% of patients (n = 27) in the first and 3.6% (n = 8) in the second trial year. The incidence of somnolence (2 cases), depression (5), syncope (1), insomnia (1), parasomnias (2), diarrhea (2), hypertension (4), and dry mouth (2) was low and sleep attacks, hallucination, dizziness, paraesthesia, and vomiting did not occur during year 2. During the second year, 21 patients (9.5%) experienced a total of 22 SAEs; none were considered to be related to trial medication. With the exception of localized osteoarthritis (4 patients), uterine leiomyoma (3), syncope (2), and goitre (2), all SAEs occurred only in single patients during the 2-year treatment period (for complete list of SAEs see Additional file 1). No deaths were reported in the 2-year study period.

**Table 2:** Incidence of adverse events (AEs) reported in ≥3% of all patients during the 2-year maintenance (safety population)

	Number of patients (%)	
	Year 1 (n = 290)	Year 2 (n = 220)
Patients with AEs	218 (75.2)	131 (59.6)
Number of AEs	630	263

Most frequent AEs^a^		
Any application site disorder^b^	100 (34.5)	36 (16.4)
Nasopharyngitis	25 (8.6)	9 (4.1)
Back pain	20 (6.9)	10 (4.5)
Erythema	19 (6.6)	2 (0.9)
Nausea	14 (4.8)	2 (0.9)
Pruritus	11 (3.8)	2 (0.9)
Insomnia	11 (3.8)	1 (0.5)
Hypertension	10 (3.4)	4 (1.8)
Fatigue	9 (3.1)	5 (2.3)
Bronchitis	9 (3.1)	5 (2.3)
Sleep disorder	9 (3.1)	5 (2.3)
Patients with serious AEs	21 (7.2)	21 (9.5)
Number of serious AEs	24	22

^a ^Incidence in ≥3% of all patients. ^b ^Includes preferred terms application site erythema, application site reaction, application site pruritus, and other related terms with lower frequency (e.g., inflammation)

There were no major changes from double-blind baseline in any of the laboratory parameters including total iron and ferritin levels. There were no patients with any post-baseline QTc ≥500 ms or post-baseline increase in QTc ≥60 ms in the ECG during the trial period.

### Tolerability outcomes

The 'daytime tiredness' score (RLS-6 scales) improved from a double-blind baseline score of 4.8 ± 2.6 by -2.6 ± 3.1 in the first year and by -2.2 ± 3.0 in the second year resulting in a total score of 2.5 ± 2.4 after 2 years of rotigotine treatment (Table 3; negative changes in the table refer to improvements). The total ESS score improved from 6.7 ± 5.2 at double-blind baseline by -0.6 ± 4.6 after 2 years (Table 3).

**Table 3:** Improvement in efficacy over a 2-year maintenance treatment with transdermal rotigotine: Mean change from baseline; mean ± SD or number (%)

	Baseline of double-blind study		Change at end of year 1 (as observed)		Change at end of year 1 (LOCF)	Change at end of year 2 (as observed)		Change at end of year 2 (LOCF)
	N	Score	N	Score	Score	N	Score	Score
IRLS total score	295	27.8 ± 5.9	220	-18.8 ± 8.8	-17.4 ± 9.9	190	-17.2 ± 9.2	-15.4 ± 10.3
RLS-6								
Satisfaction with sleep	293	7.2 ± 2.5	216	-4.5 ± 3.4	-4.1 ± 3.5	190	-4.3 ± 3.3	-3.7 ± 3.4
Severity at bedtime	293	6.0 ± 3.0	215	-4.6 ± 3.2	-4.2 ± 3.3	189	-4.0 ± 3.1	-3.8 ± 3.2
Severity during the night	294	6.8 ± 2.7	216	-5.2 ± 3.0	-4.9 ± 3.1	190	-4.9 ± 3.0	-4.5 ± 3.2
Severity during the day when resting	293	4.9 ± 2.6	216	-3.4 ± 2.6	-3.4 ± 2.7	190	-2.9 ± 2.7	-2.9 ± 3.0
Severity during the day when active	294	1.9 ± 2.0	217	-1.4 ± 1.9	-1.5 ± 2.0	190	-1.2 ± 2.0	-1.2 ± 2.2
Daytime tiredness or sleepiness	294	4.8 ± 2.6	217	-2.8 ± 2.9	-2.6 ± 3.1	190	-2.4 ± 2.7	-2.2 ± 3.0
CGI-1 (severity of illness)	295	5.1 ± 0.9	217	-2.9 ± 1.2	-2.8 ± 1.3	191	-2.8 ± 1.2	-2.6 ± 1.4
QoL-RLS total score	273	30.2 ± 10.4	200	-19.1 ± 12.2	-17.7 ± 13.2	178	-17.7 ± 12.8^a^	-15.8 ± 13.8^a^
ESS total score	289	6.7 ± 5.2	215	-1.5 ± 4.5	n.d.	185	-0.8 ± 4.6	-0.6 ± 4.6

^a ^after 21 months of maintenance

Based on CGI tolerability rating, a total of 70% of the 220 patients completing year 1 and 71% of the 191 patients completing the second trial year were not impaired by side effects at all; 28.1% of the year 1 completers and 28.3% of the year 2 completers reported that side effects did not significantly interfere with their daily activities. At the end of the 2-year period, a 'significant interference' and 'side effects outweighing efficacy' were reported in 13% and 6.8%, respectively, of all patients who had entered the trial.

Rotigotine tolerability after 2 years of treatment was rated as 'very good' or 'good' by 77% of all patients (n = 295). Seventeen patients (5.8%) described tolerability as 'very bad'.

Patch adhesiveness and ease of removal were considered moderate (15%/4.5%), good (50%/58%), or excellent (31%/34%) by most patients treated in year 2 (n = 220).

### Efficacy

**Figure 2:**
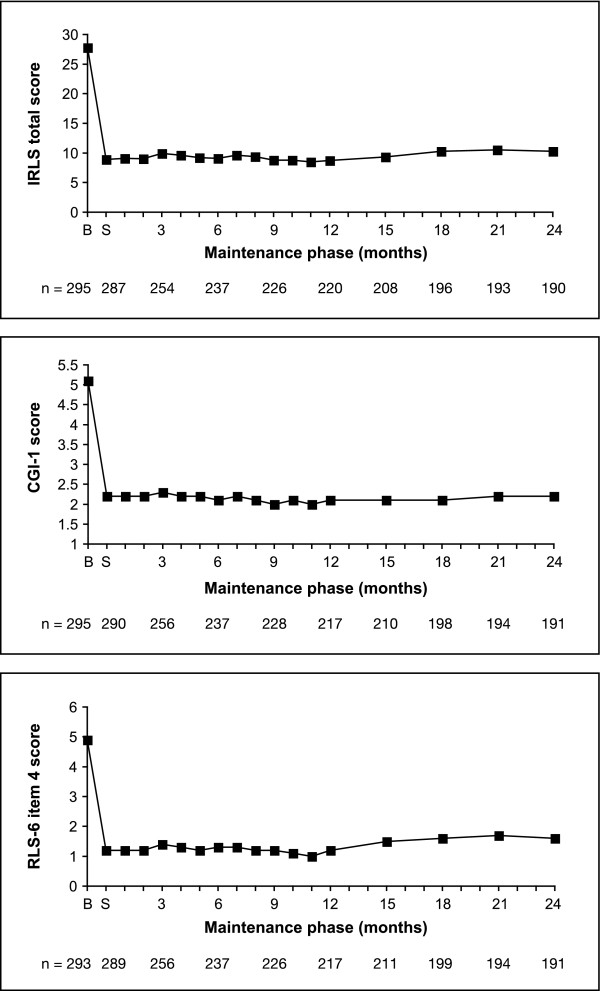
**Mean IRLS total score, CGI item 1 (severity of illness), and RLS-6 item 4 (severity during the day at rest) scores over 2 years of rotigotine maintenance treatment (data as observed)**. B, baseline; CGI, Clinical Global Impression; IRLS, International RLS Severity Scale; RLS, restless legs syndrome S, start of maintenance phase.

**Figure 3:**
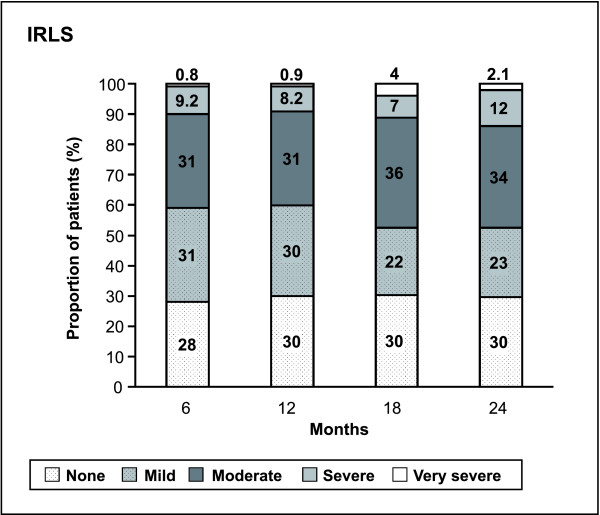
**Changes in IRLS severity over 2 years of rotigotine maintenance treatment (data as observed)**.

Rotigotine transdermal patch rapidly improved the IRLS severity rating; the baseline sum score of 27.8 ± 5.9 was reduced by 18.8 ± 8.6 during the 4-week titration phase of the open-label trial (Figure 2). This result is comparable to the data recorded during rotigotine titration in the preceding double-blind, placebo-controlled trial [13]. Figure 3 shows the changes in IRLS severity over 2 years of rotigotine treatment. At month 12 and month 24, mean IRLS sum scores of 8.7 ± 8.0 and 10.3 ± 9.3, respectively, were calculated. Mean reduction after 2 years of rotigotine maintenance treatment was 15.4 ± 10.3 for all patients entering this extension trial (n = 295, LOCF) and 17.2 ± 9.2 for all patients completing the 2-year period (n = 190). CGI-1 and RLS-6 daytime at rest scores closely follow this pattern (Figure 2). All other efficacy variables also improved over the 2-year period (Table 3) and remained stable during the second year when compared with the year 1 results [16]. Responder rates indicate that the majority of patients benefited from rotigotine treatment (Table 4). Overall, 30% of all patients who completed the 2-year treatment were free of symptoms according to IRLS (total score = 0) and 30% were free of symptoms according to CGI-1 (not ill at all). Therapeutic efficacy of rotigotine was rated as 'good' to 'very good' in 95% (n = 209) of year 1 completers and 89% (n = 170) of year 2 completers.

**Table 4:** Improvement in efficacy over a 2-year maintenance treatment with transdermal rotigotine: responder and remitter rates compared with baseline

	End of year 1		End of year 2	
	As observed (n = 217)	n = 295	As observed (n = 190)	n = 220
IRLS total score				
Responder (≥50% improvement)	75%	68%	65%	79%
Remitter (total score ≤10)	60%	54%	53%	62%
Symptom-free (total score = 0)	30%	25%	30%	32%
CGI-1 responder (≥50% improvement)	79%	72%	75%^a^	65%
CGI-2 responder ('much' or 'very much' improved)	97%	89%	95%^a^	85%

^a ^n = 191

CGI, Clinical Global Impression; ESS, Epworth Sleepiness Scale; IRLS, International RLS Severity Scale; QoL, quality of life; RLS, restless legs syndrome

Range of scales: IRLS: 0 to 40 (= severe), RLS-6: 0 to 10 (= severe); CGI-1: 1 to 7 (= extremely severe), QoL-RLS: 0 to 60 (= severe impairment)

## Discussion

Rotigotine transdermal patch is the first transdermally applied dopamine agonist for the treatment of symptoms of RLS and covers a 24-h period with a single administration. The continuous rotigotine delivery seems to translate into long-term benefits for patients moderately to severely affected by RLS. Over a period of 2 years, a rotigotine dose range of 0.5 mg/24 h to 4 mg/24 h provided sustained relief from symptoms with a very good tolerability and a favorable safety profile.

Rotigotine dose levels remained quite stable from the first to the second trial year with only a small dose increase of 2.9% for a mean daily dose of 2.93 mg/24 h after 2 years of treatment. The most frequently applied rotigotine dose at the end of 2 years was 4 mg/24 h (44.5% of patients); 13.5% were sufficiently treated with a low dose of 0.5 or 1.0 mg/24 h. In the preceding 6-week dose-finding trial with a fixed dosing schedule, the 4 mg/24 h dose did not show an additional benefit compared with 3 mg/24 h over the 6-week treatment period. The likely therapeutic window for the treatment of idiopathic moderate to severe RLS was thus established as ranging from 1 mg/24 h to 3 mg/24 h [13]. The distribution of individual rotigotine doses in this trial supports the opinion of most RLS experts that dose selection has to be adjusted according to efficacy and tolerability experiences of each individual patient and dosages of rotigotine both lower or higher than the recommended doses of 1 mg/24 h or 3 mg/24 h may be appropriate during long-term rotigotine therapy.

Discontinuation rates owing to a lack or loss of efficacy through 2 years of rotigotine treatment were low (5.8%). No comparable data for this length of study duration are available for other dopamine agonists. Discontinuation rates for studies of shorter duration are as follows: 3.3% within 6 months [22] and 7.9% within 30 weeks [23] of treatment with cabergoline, and 3.5% within 52 weeks of treatment with ropinirole [8]. Open-label treatment with pramipexole for 26 weeks did not lead to withdrawal for efficacy reasons [7].

Augmentation, the main complication of long-term dopaminergic treatment in RLS [24], is mainly characterized by an earlier appearance of RLS symptoms in the day and an overall increase in the severity of RLS symptoms. Augmentation was given as the reason for seven dose adjustments - a low occurrence of 2.4% over 2 years. In the absence of any validated diagnostic tool for the diagnosis of augmentation at the start of the study, a detailed retrospective analysis by an expert panel is planned following completion of the full 5-year observation period.

Known dopaminergic side effects such as nausea and fatigue occurred mainly during titration and the first study year [16]. Incidences during the first year were similar to the rates reported in open-label long-term trials for cabergoline (6 months) [22] and pramipexole (26 weeks) [7] but lower than for ropinirole (52 weeks) [8]. During the second year, only two cases of nausea and five cases of fatigue were documented, and vomiting was not reported as an AE. The incidence of somnolence was also lower in year 2 (two cases compared with five cases in year 1), and hallucinations, dizziness, and sleep attacks were not reported.

As expected with a transdermal application, skin reactions were common with higher rates in the first (34.5%, 100/290 patients) compared with the second (16.4%, 36/220) trial year. Most cases were mild to moderate in intensity. However, a proportion of patients discontinued owing to application site problems: 27 patients left the study in year 1 and 8 patients in year 2. Similar or higher discontinuation rates of patch treatments were reported for other indications [25,26].

Investigators noted 'much' and 'very much' improvement of RLS in 95% of all patients who had entered this open-label extension. The severity of the condition as measured with the IRLS already improved during the titration period, remained stable during the first trial year [16] and only slightly increased during the second year. More than half of the patients completing the study were regarded as remitters (53%) and 30% were symptom-free after 2 years of rotigotine treatment. Responder rates underline the sustained, long-term benefit of the rotigotine transdermal patch. The improvement in QoL paralleled by the improvement of RLS symptoms during the night as well as during the day (as shown in the RLS-6 scales) supports the benefit of delivery of rotigotine over 24 hours in the treatment of RLS.

The good long-term efficacy and safety profile of transdermal rotigotine resulted in high retention rates: after 2 trial years it was 66%, and 87% of the patients entering the second year completed 2 years of maintenance. Compliance in the second year was still 92% and the majority of the patients rated tolerability and efficacy of the treatment as 'good' or 'very good'. Treatment satisfaction is also underlined by the QoL-RLS score which was improved by 50% at the end of the 2-year follow-up from baseline of the double-blind trial [13].

In interpreting these long-term open-label data, it is important to note the limitation presented by the absence of a comparator arm; however, inclusion of a placebo group for 2 years of follow-up is inappropriate both practically and ethically. In the preceding 6-week, double-blind study a reduction in IRLS score of 9.2 points from baseline was observed in the group of patients randomized to placebo [13]. The 15- to 17-point reduction observed with open-label rotigotine is consistent with the reductions achieved with rotigotine doses of 1-4 mg/24 h during the double-blind study. Although these open-label data support the assumption of a symptomatic benefit over a 2-year period, the net effect on IRLS reduction with rotigotine is likely less than the 15-17 points reported here.

Putting both efficacy and tolerability results into perspective, the 2-year data of the rotigotine trial indicate a selection process: patients who do not tolerate either the dopamine agonist or the patch administration, or both, generally discontinue treatment during the first year of therapy. Perceived loss of efficacy is not a major issue during long-term therapy with rotigotine in the great majority of the patients. Thus a high number of patients who stayed on therapy benefited from rotigotine as expressed in the fact that 30% of patients (57 of 190 patients) were free of symptoms after 2 years.

## Conclusions

The findings of this trial suggest that long-term administration of rotigotine transdermal patch is generally well tolerated and an efficacious treatment option for patients with moderate to severe RLS. The treatment markedly improved RLS symptoms and quality of life of the patients.

## Competing interests

The study was sponsored by Schwarz Biosciences GmbH, a member of the UCB Group of Companies, delegated by Schwarz Pharma Ltd., Ireland. BH received a grant, consultancy honoraria, and honoraria for serving on scientific advisory boards from the sponsor. BH has also received consultancy and/or speakers honoraria from Boehringer Ingelheim, GlaxoSmithKline, Jazz, Novartis, Sanofi, and Lundbeck; honoraria for serving on scientific advisory boards from Boehringer Ingelheim, Sanofi, Cephalon, Nycomed, and Lundbeck; and royalties from Cambridge University Press. She is currently employed by Innsbruck Medical University.

WHO received consultancy honoraria, and honoraria for serving on scientific advisory boards from the sponsor. WO has also received honoraria for serving on the scientific advisory boards and/or for speaking engagements from Bayer-Schering, Bioprojet, Boehringer Ingelheim, Desitin, GlaxoSmithKline, Lundbeck, Meda Pharmaceuticals International, Merck-Serono, Neurosearch, Novartis, Orion Pharma, Proteosys, Schering Plough, Schwarz Pharma Neuroscience (UCB), Solvay Pharmaceuticals, Synosia, and Teva. He received scientific grants from the German Ministry of Education and Health and owns Roche 100 stock.

KSK has received honoraria for serving on scientific advisory boards and honoraria for speaking engagements from UCB and Schwarz Pharma. KSK has also received honoraria for serving on the scientific advisory boards for Boehringer Ingelheim, Orion, Mundipharma, Pfizer, and Synosia, and she received honoraria for speaking engagements sponsored by Boehringer Ingelheim.

PG received honoraria for speaking engagements and honoraria for serving on scientific advisory boards from the sponsor. PG has also received honoraria for speaking engagements by Boehringer Ingelheim, Servier, Lundbeck, Cephalon, EISAI, ResMed, and MPV Truma. He is currently employed with Medizinische Einrichtungen des Bezirks Oberpfalz, Department of Psychiatry.

HB received consultancy honoraria and honoraria for serving on scientific advisory boards from the sponsor. HB has also received consultancy honoraria from Boehringer Ingelheim, GlaxoSmithKline and MSD; and honoraria for serving on scientific advisory boards from Boehringer Ingelheim and GlaxoSmithKline.

DGB received compensation for speaking and for consulting services from the sponsor. DGB has also received compensation for speaking and/or consulting services from Boehringer Ingelheim, GlaxoSmithKline, Pfizer, Lundbeck, Sanofi-Aventis, and Jazz Pharma.

CT received compensation for consulting services from the sponsor. CT has also received compensation for consulting services from Boehringer Ingelheim, Solvay, GlaxoSmithKline, Lundbeck, Vito Pharma, Axxonis Pharma, and Cephalon.

WP received consultancy honoraria and honoraria for serving on scientific advisory boards from the sponsor. WP has also received honoraria for consultancy and lecture fees from Teva, Novartis, GSK, Boehringer-Ingelheim, UCB/Schwarz Pharma, and Orion Pharma in relation to clinical drug development programmes for PD.

ES is an employee of the study sponsor.

RK received honoraria for services on scientific advisory boards from the sponsor. RK has also received honoraria for services on scientific advisory boards from Axxonis Pharma, Pfizer and Strathmann. He is currently an employee of ReSearch Pharmaceutical Services Inc, USA.

## Authors' contributions

BH participated in study conception and design, the execution of the research project, data interpretation, manuscript writing, and manuscript review and critique. WO participated in study conception and design, execution of the research project, data interpretation, and critical revision of the manuscript. KSK participated in study conception and design, data collection, interpretation of results, and reviewing of the manuscript. PG participated in the execution of the research project, interpretation of data, and critical revision of the manuscript. HB participated in study conception and design, the execution of the research project, data interpretation, and preparation of revised versions/review. DGB participated in study conception and design, data collection, the interpretation of data and critical revision of the manuscript. CT participated in study conception and design, the execution of the research project, interpretation of data, and critical revision of the manuscript. WP participated in data collection, the interpretation of data and critical revision of the manuscript. ES participated in the study design, execution of the research project, interpretation of data, and critical revision of the manuscript. RK participated in protocol development, study conduct, interpretation of the data, and in manuscript writing. All authors read and approved the final version of the manuscript.

## Appendix

### Rotigotine SP710 Study Group

Austria: W. Poewe/B. Högl (Innsbruck), B. Saletu (Vienna)

Germany: H. Benes (Schwerin), B. Bergtholdt (Berlin), R. Bodenschatz (Mittweida), P. Clarenbach (Bielefeld), I. Eisensehr (Munich), P. Franz (Berlin), P. Geisler (Regensburg), U. Hegerl (Nuremberg), W. Käfferlein (Bamberg), M. Lang, (Ulm) G. Karlbauer (Munich), S. Krämer (Berlin), I. Maier (Tuttlingen), G. Mayer (Schwalmstadt-Treysa), W. Oertel/K. Stiasny-Kolster (Marburg), C. Öhlwein (Gera), I. Peglau (Berlin), K. Sallach (Gelsenkirchen), K. Schlinsog (Halle), T. Schwerdtfeger (Naumburg), A. Schulze (Berlin), A. Siever (Oldenburg), K. Sigel (Munich), H. Sommer (Koethen), K. Tinschert (Jena), C. Trenkwalder (Kassel/Göttingen), B. Veit (Neubrandenburg), R. Warmuth (Berlin)

Spain: E. Estevil (Barcelona), D. Garcia-Borreguero (Madrid), F. Puertas (Valencia)

## Pre-publication history

The pre-publication history for this paper can be accessed here:

http://www.biomedcentral.com/1471-2377/10/86/prepub

## Supplementary Material

Additional file 1 **Further SAEs occurring in single patients only**. This file contains a list of the SAEs which occurred in single patients only during the 2-year treatment period Click here for file
