# Supplementary material for: Treatment of moderate to severe restless legs syndrome: 2-year safety and efficacy of rotigotine transdermal patch
Source: BMC Neurol. 2010 Sep 28;10:86. doi: 10.1186/1471-2377-10-86 (PMC2958158; doi:10.1186/1471-2377-10-86)
Supplement: Additional file 1 — Further SAEs occurring in single patients only. This file contains a list of the SAEs which occurred in single patients only during the 2-year treatment period [file 1471-2377-10-86-S1.DOC]

**Supplemental material**

**Results**

**Further SAEs occuring in single patients only**

Lymphadenopathy, myocardial infarction, pericardial hemorrhage, bradycardia, tachycardia, gastric ulcer hemorrhage, inguinal hernia, nausea, hernia, cyst, peripheral oedema, acute cholecystitis, gastroenteritis, perianal abscess, chronic pyelonephritis, meniscus lesion, ankle fracture, incisional hernia, facial bones fracture, increased blood pressure, toe deformity, intervertebral disc protrusion, colon adenoma, plasmacytoma, polyneuropathy, cerebellar infarction, tension headache, migraine, dizziness, restless legs syndrome (hospitalization for diagnostic reasons, sleep lab), abortion, bladder operation, limb operation, hysterectomy, arterial occlusive disease, vascular occlusion, deep vein thrombosis, varicose vein.
